# Bridging the Gap in AL and ATTR Cardiac Amyloidosis: Integrating Histopathology, Biomarkers, and Multimodal Imaging for Subtype-Specific Diagnosis

**DOI:** 10.31083/RCM42153

**Published:** 2026-01-23

**Authors:** Karim Ali, Mohamed K. Awad, Hussain Majeed, Mohamed S. Amer, Ahmad Alayyat, Ahmed E. Ali, Abdelrahman Ali, Ahmed Sami Abuzaid

**Affiliations:** ^1^Department of Internal Medicine, Hennepin County Medical Center, Minneapolis, MN 55415, USA; ^2^Department of Critical Care, Faculty of Medicine, Ain Shams University, 11646 Cairo, Egypt; ^3^Department of Internal Medicine, Northeast Georgia Medical Center, Gainesville, GA 30501, USA; ^4^Department of Cardiology, El Mansoura International Hospital, 35516 Mansoura, Egypt; ^5^Department of Internal Medicine, Hamilton Medical Center, Dalton, GA 30720, USA; ^6^Department of Internal Medicine, Crestwood Medical Center, Huntsville, AL 35801, USA; ^7^Department of Cardiology, University of Texas Medical Branch, Galveston, TX 77555, USA; ^8^Alaska Heart and Vascular Institute, University of Alaska, Anchorage, AK 99571, USA

**Keywords:** cardiac amyloidosis, molecular pathophysiology, biomarker-based diagnosis, transthyretin amyloidosis, light-chain amyloidosis

## Abstract

Cardiac amyloidosis (CA) represents an increasingly recognized but historically underdiagnosed cause of restrictive cardiomyopathy and heart failure. CA is now understood to be more prevalent, particularly in older adults, as advancements in imaging and biomarker technologies have improved detection. The disease results from the misfolding of precursor proteins, primarily immunoglobulin light chains (in light chain (AL) amyloidosis) or transthyretin (in transthyretin (ATTR) amyloidosis), into insoluble fibrils that deposit in myocardial tissue. These deposits cause structural and functional cardiac impairment through both physical infiltration and cytotoxic mechanisms, leading to diastolic dysfunction, arrhythmias, and progressive heart failure. Understanding the molecular basis of amyloid formation and deposition has revealed subtype-specific mechanisms of toxicity and tissue tropism, highlighting the central role of protein instability, proteolytic cleavage, and oxidative stress in disease progression. Furthermore, increasing awareness of phenotypic variability and sex- or ethnicity-based diagnostic disparities has called for earlier recognition and differentiation of CA subtypes. Diagnostic precision is enhanced by a multimodal approach incorporating histopathology, biomarker staging, and advanced imaging techniques such as echocardiography, cardiac magnetic resonance, and nuclear scintigraphy. This review addresses our contemporary understanding of the molecular mechanisms, pathophysiologic cascade, and diagnostic evolution of AL and ATTR CA, emphasizing clinical progress. By delineating the biological mechanisms and tools for early identification, this paper aims to strengthen the framework for diagnosing and managing a disease that was once overlooked but is now at the forefront of modern cardiovascular medicine.

## 1. Introduction

Cardiac amyloidosis (CA) is an underrecognized cause of heart failure characterized by the extracellular deposition of misfolded amyloid fibrils within the myocardium, leading to restrictive cardiomyopathy. As awareness of the disease grows, accurate diagnosis remains a clinical challenge due to its overlap with other cardiac conditions. Histopathologic evaluation remains the gold standard for diagnosis, while biomarker-based approaches offer non-invasive, accessible tools for early detection and prognosis. This review explores the evolving landscape of CA diagnostics, focusing on tissue characterization techniques and the clinical utility of natriuretic peptides and troponins, with particular emphasis on the two most clinically relevant subtypes, light chain (AL) and transthyretin (ATTR) amyloidosis, aiming to integrate pathological and biomarker-based strategies for optimal patient care.

## 2. Epidemiology, Sex Disparities, and the Diagnostic Challenge of Cardiac Amyloidosis

CA is frequently misdiagnosed, leading to significant delays in diagnosis, which can adversely affect patient outcomes. In a survey of over 500 patients with AL amyloidosis, 37% of whom had cardiac involvement, the average time from the first symptoms to a diagnosis was 2 years. A notable proportion of patients (31.8%) saw at least five different physicians before being diagnosed with amyloidosis. Although cardiologists were consulted the most, they were only responsible for the diagnosis in 18.7% of cases. Similar trends were observed in ATTR-CA, albeit in smaller sample sizes, where fewer than half of patients were diagnosed within six months, with cardiologists making the diagnosis in most cases [1].

The optimal diagnosis and management of cardiac amyloidosis necessitate a multidisciplinary approach, involving expertise from cardiology, neurology, nephrology, and hematology. However, specialized amyloidosis teams remain limited. Over a decade between 2000 and 2012, the prevalence rate of cardiac amyloidosis has raised from 8 to 17 per 100,000 person-years due the improvement of survival and the advances in diagnostics [2].

ATTRwt cardiac amyloidosis predominantly affects adults over the age of 60 with a strong male predominance. The estimated prevalence is over 10% and frequently diagnosed as heart failure with preserved ejection fraction (HFpEF). At least 10% of patients with aortic stenosis may have ATTRwt amyloidosis [3, 4, 5]. Autopsy studies have shown that 25% of adults over the age of 80 have transthyretin amyloid deposits in the myocardium [6]. Among patients with heart failure with preserved ejection fraction (HFpEF), amyloid deposits are found in 32% of individuals aged over 75, compared to 8% in those under 75 years of age [7, 8].

Both forms of transthyretin-related cardiac amyloidosis (ATTR-CM) exhibit a clear male predominance, with approximately 80% of ATTRwt cases and 70% of ATTRv cases occurring in men. In contrast, light chain cardiac amyloidosis (AL-CM) does not show a significant sex disparity, with reported male-to-female ratios ranging from 1:1 to 1.5:1 [9].

​It was found that only 9% of patients diagnosed with wild-type transthyretin amyloidosis (ATTRwt) before death were women, while 31% of post-mortem diagnoses were in women, underscores a significant sex-based diagnostic disparity. This discrepancy suggests that ATTRwt may be underdiagnosed in women during their lifetime [10]. This underdiagnosis is further supported by findings from one of the largest autopsy series conducted by Hodkinson and Pomerance, who reported amyloid deposits in nearly 50% of unselected individuals over the age of 60, with a prevalence of 56% in women compared to 37.5% in men. Although women in that study more commonly had atrial amyloid, likely due to atrial natriuretic factor deposition, 29% of them also had ventricular amyloid, suggesting that clinically significant cardiac involvement in women may be more prevalent than previously recognized [11].

A study examining sex-related differences in wild-type transthyretin cardiac amyloidosis (ATTRwt-CM) found that although women were diagnosed at an older age and exhibited less left ventricular hypertrophy, the prevalence of atrial fibrillation (AF) did not significantly differ between genders, 49% in females versus 55% in males (*p* = 0.53). These results suggest that, despite differences in clinical presentation, the risk of arrhythmias such as AF is similar in both men and women with ATTRwt-CM [12]. Gender was not statistically significant between the AF (Atrial Fibrillation) and non-AF groups in patients with light-chain or transthyretin cardiac amyloidosis. This indicates that there was no significant difference in the gender distribution between the two groups, suggesting that gender did not have a measurable impact on the presence of AF in this cohort of patients [13].

AL-CA typically manifests at an earlier age compared to ATTRwt-CA, with a slight male predominance. While its exact prevalence is not well-defined, it is considerably rarer than ATTR-CA. AL-CA is estimated to affect 8 to 12 individuals per million [14, 15], with approximately 3000 new cases of AL amyloidosis diagnosed annually in the United States. Of these, 30–50% present with symptomatic cardiac involvement, and 10–15% are associated with multiple myeloma [16].

Even in patients where other organ systems are primarily affected, cardiac involvement remains the strongest predictor of poor prognosis in AL amyloidosis [17] and contributes to approximately 75% of deaths [18]. The onset of heart failure symptoms represents a pivotal moment in disease trajectory, after which survival sharply declines typically to less than six months in AL CA without treatment to three to five years for TTR [5, 19].

### 2.1 Genetic and Ethnoracial Variability

Transthyretin (TTR) is a plasma protein consisting of 127 amino acids that is made up of four non-covalently bound subunits enriched in β-sheet structures, which together create two binding sites for thyroxine (T4). The *TTR gene* is located on chromosome 18 and comprises four exons and five introns. Over 120 mutations have been identified in the *TTR gene*. However, only a small number of these mutations, including *Val30Met*, *Thr60Ala*, *Ser77Tyr*, and *Val122Ile*, are responsible for the majority of hereditary ATTR cases globally which are inherited as autosomal dominant with variable penetrance [3, 20, 21, 22].

Both the *Thr60Ala* and *Val122Ile* mutations are strongly associated with cardiac involvement in ATTR amyloidosis. *Thr60Ala* is more frequently observed in populations from the United Kingdom and the United States, while *Val122Ile* is the most prevalent TTR mutation worldwide, particularly notable for its association with late-onset cardiac amyloidosis that closely mimics the clinical presentation of the wild-type form [3, 23]. In addition, rarer mutations such as *Leu111Met*, identified in Denmark [24] and *Ile68Leu*, reported in Italy [25], have also been linked to cardiac amyloidosis. The *Val30Met* mutation is particularly prevalent in Portugal, Spain, France, Japan, Sweden, and in countries with descendants from these regions, such as Brazil, where it is the most common cause of hereditary transthyretin amyloidosis (hATTR) [26, 27].

The genetic basis for cardiac amyloidosis was first proposed by the Los Angeles County study, which reviewed 52,370 autopsies. The study found that the prevalence of cardiac amyloidosis was significantly higher among African Americans (1.6%) compared to White Americans (0.42%), despite the fact that other forms of amyloidosis were less common in African Americans [28].

In the United States, wild-type transthyretin amyloidosis (ATTRwt) accounts for approximately 48% of all ATTR cases, followed by the *Val122Ile* mutation, which contributes to around 23%. The *Val122Ile* is prevalent in 3.4% of African Americans, making it the most common TTR mutation in the U.S, followed by *Thr60Ala*, often referred to as the Appalachian mutation, predominantly seen in individuals of Irish descent [29, 30, 31]. *Thr60Ala* is the most common mutation in the United Kingdom, where it predominantly affects Caucasian individuals [23].

Outside the United States, ATTRwt accounts for only 5% of cases, whereas 76% of patients have hereditary ATTR (ATTRm), predominantly due to the *Val30Met* mutation. Demographic patterns also differ by mutation type: ATTRwt patients are primarily Caucasian (89.4%), while those with the *Val122Ile* variant are predominantly of African descent (86.8%) [5, 29, 31].

### 2.2 Beyond the Heart: Recognizing Multisystemic Clues

Although cardiac involvement is common in amyloidosis, it is rarely the sole manifestation. As such, recognizing clinical “red flags” suggestive of systemic amyloidosis is essential to raising the index of suspicion and facilitating earlier diagnosis of CA [32].

Wild-type transthyretin amyloidosis (ATTRwt) is a systemic disorder in which cardiac involvement is typically the dominant clinical manifestation, although other organs may also be affected subclinically. Importantly, orthopedic manifestations often precede the development of overt cardiac amyloidosis (ATTR-CA) by several years [21]. These may include carpal tunnel syndrome, lumbar spinal stenosis, biceps tendon rupture, and hip or knee arthroplasty. While the heart is usually the primary organ involved at the time of diagnosis, awareness of these earlier musculoskeletal signs can aid in the timely identification of ATTRwt amyloidosis [33, 34, 35, 36].

Amyloid deposits have been identified in 7% to 8% of patients with carpal tunnel syndrome based on tenosynovial tissue biopsy [37]. Bilateral Carpal tunnel syndrome is recognized as one of the red flags for systemic amyloidosis, particularly transthyretin cardiac amyloidosis, and its presence should prompt further screening for cardiac involvement [38]. While the majority of patients with both wild-type transthyretin amyloidosis (ATTRwt) and hereditary ATTR due to the *Val122Ile* mutation (ATTRm *Val122Ile*) present with cardiac symptoms, rhythm disturbances including atrial fibrillation and sinoatrial block are more commonly observed in ATTRwt (65.5%) compared to *Val122Ile* ATTRm (32%).

Data from the Transthyretin Amyloid Outcomes Survey (THAOS) further highlight phenotypic differences between these subtypes. Patients with *Val122Ile* ATTRm exhibit higher rates of gait instability (18.1% vs. 6.7%), gastrointestinal symptoms such as diarrhea and/or constipation (18.9% vs. 8.6%), neuropathic pain (33.8% vs. 12%), and urinary incontinence (4.1% vs. 0.6%) compared to those with ATTRwt, indicating greater involvement of the peripheral and autonomic nervous systems [31].

Additionally, the *Thr60Ala* mutation (also known as the Appalachian mutation) is reported as the third most common cause of ATTR cardiac amyloidosis in the United States, and is similarly associated with significant peripheral and autonomic nervous system involvement [39]. Hereditary transthyretin amyloidosis (ATTRv) frequently presents with sensorimotor polyneuropathy, especially in association with neuropathy-predominant mutations such as *Val30Met*. However, several mutations are associated with cardiac-dominant phenotypes, including Val122Ile, Leu111Met, and *Ile68Leu*, which primarily manifest with features of cardiac amyloidosis rather than peripheral neuropathy [32].

Approximately 5% to 10% of patients with AL amyloidosis have concurrent overt multiple myeloma, and a similar proportion of multiple myeloma patients develop AL amyloidosis. AL amyloidosis is estimated to be about one-tenth as common as multiple myeloma, with an annual age-adjusted incidence of approximately 10.5 cases per million person-years in the United States [40].

In AL amyloidosis, cardiac involvement frequently occurs alongside renal, neurological, and dermatological manifestations, reflecting the multisystemic nature of the disease. The co-occurrence of these organ involvements is common at presentation and contributes to the clinical heterogeneity and complexity of diagnosis. AL amyloidosis most commonly affects the kidneys, typically presenting with nephrotic syndrome, while cardiac involvement represents the second most frequent clinical manifestation [14].

Traditionally, reviews have emphasized that cardiac AL amyloidosis rarely occurs in isolation, citing that isolated cardiac disease is present in fewer than 5% of cases. However, this estimate may underrepresent the true burden, as clinically isolated AL cardiac amyloidosis is likely underdiagnosed. This underestimation is partially due to the rapid disease progression and high early mortality in patients who remain undiagnosed. With the increasing use of advanced diagnostic tools in the evaluation of heart failure, earlier identification of cardiac amyloidosis is improving, possibly leading to better detection of cases that were previously overlooked [14].

### 2.3 Determinants of Tissue Tropism: Why the Heart?

The organ dysfunction seen in both AL and ATTR cardiomyopathy cannot be fully explained by the mere displacement of normal parenchymal tissue by amyloid deposits [41]. Instead, it involves a highly complex aggregation process of amyloid proteins within target organs [42].

In addition, amyloid deposition in specific organ tissues is likely influenced by a combination of factors, including elevated local protein concentrations, acidic pH, and the presence of fibril seeds. Specific interactions with tissue components such as glycosaminoglycans or cell surface receptors may play a critical role in promoting localized aggregation [43]. In ATTR, this aggregation is primarily driven by two adhesive segments that form the F and H β-strands in the native transthyretin (TTR) structure. When TTR dissociates into monomers, these segments become exposed, facilitating the stacking into steric zipper spines that characterize amyloid fibrils [44].

In ATTRv, the specific site of amino acid substitution significantly influences the tendency for amyloid deposits to primarily accumulate in either the peripheral nervous system or cardiac tissue, resulting in distinct disease phenotypes. For example, the *Val122Ile (p.Val142Ile)* TTR variant is predominantly associated with cardiomyopathy, while variants like *Val30Met (p.Val50Met)* are more commonly linked to neuropathy. The fragmentation of the TTR monomer into smaller fibrils may also play a role in determining where amyloid deposits accumulate and in influencing the disease’s penetrance [45].

In ATTRv caused by the *Val30Met (p.Val50Met)* variant, full-length TTR fibrils are typically observed in early-stage disease, which is characterized by predominant axonal polyneuropathy and rare cardiac involvement. However, in later-onset cases, fibrils often consist of a mixture of full-length and truncated TTR, with patients presenting with significant cardiac involvement alongside peripheral neuropathy. Similarly, in ATTRwt which presents late, amyloid deposits consistently contain both fragmented and full-length TTR fibrils [46].

In AL amyloidosis, it is hypothesized that organ tropism may result from polymorphisms in the variable region gene, which could influence interactions between the light chain (or its fragments) and tissue components such as collagen, lipids, and glycosaminoglycans [47]. A recent study on AL amyloidosis highlighted the role of immunoglobulin light-chain variable (Ig VL) regions and their corresponding germline genes in determining organ tropism. Specific Ig VL clones are linked to distinct patterns of organ involvement, for instance, some are associated with renal tropism, while others preferentially target the heart and are often linked to multisystem disease. This variability in clinical presentation has also been connected to the presence of N-glycosylation in kappa light chains, which appears to enhance their amyloidogenic potential [43, 48].

It was demonstrated that light chains derived from the LV1-44 germline gene preferentially deposit in cardiac tissue, contributing to cardiac-dominant AL amyloidosis [49]. Similarly, the 1c, 2a2, and 3r germline genes are associated with dominant cardiac and multisystem involvement, while the 6a gene is strongly linked to renal-predominant disease. These findings highlight the critical role of light-chain variable region germline origin in influencing organ tropism in AL amyloidosis [48].

In addition to the mechanical damage caused by amyloid fibril deposition, small soluble monomers and oligomers are highly toxic and are believed to play a significant role in cell and tissue toxicity. The direct toxic effect of circulating light chains in AL amyloidosis has been proposed to explain the discrepancies observed between myocardial amyloid fibril burden, cardiac dysfunction, and the more aggressive disease progression in AL compared to ATTR cardiac amyloidosis (ATTR-CA). Notably, both amyloid deposition and light chain proteotoxicity exhibit specific organ tropism, contributing to the varying severity of organ involvement in the disease [50].

## 3. Molecular Mechanisms and Pathophysiologic Cascade in Cardiac Amyloidosis

CA is a protein-misfolding cardiomyopathy in which normally soluble proteins misfold into insoluble, β-sheet–rich amyloid fibrils that deposit in the heart [51]. Historically regarded as a rare “zebra”, CA is now recognized with increasing frequency, owing to an aging population, improved noninvasive diagnostics (e.g., bone scintigraphy for transthyretin amyloid), and heightened clinical suspicion driven by newly available therapies [52]. CA most commonly results from either an immunoglobulin light-chain (AL) amyloidosis or a transthyretin (ATTR) amyloidosis, which together account for >95% of cases [32]. In AL-CA, a clonal plasma cell dyscrasia leads to overproduction of misfolded light chains that aggregate into cardiac amyloid fibrils [14]. In ATTR-CA, the culprit is the liver-derived transthyretin protein, which in its wild-type form destabilizes with age or, in mutant variants, is destabilized by autosomal dominant mutations [53]. In both situations, native protein subunits misfold and self-assemble into oligomers and then fibrils, which deposit in the myocardium. Co-deposited serum amyloid P components, apolipoproteins, and glycosaminoglycans may further stabilize the fibrils [52, 54]. Notably, beyond passive deposition, the amyloidogenic proteins themselves exert direct toxicity: soluble prefibrillar light chains and ATTR oligomers can infiltrate cell membranes, provoke oxidative stress, impair calcium handling, activate pro-apoptotic pathways, and ultimately induce cardiomyocyte death [52, 55]. This dual injury—physical infiltration plus biochemical toxicity—sets the stage for a unique cardiomyopathy.

**Fig. 1.**
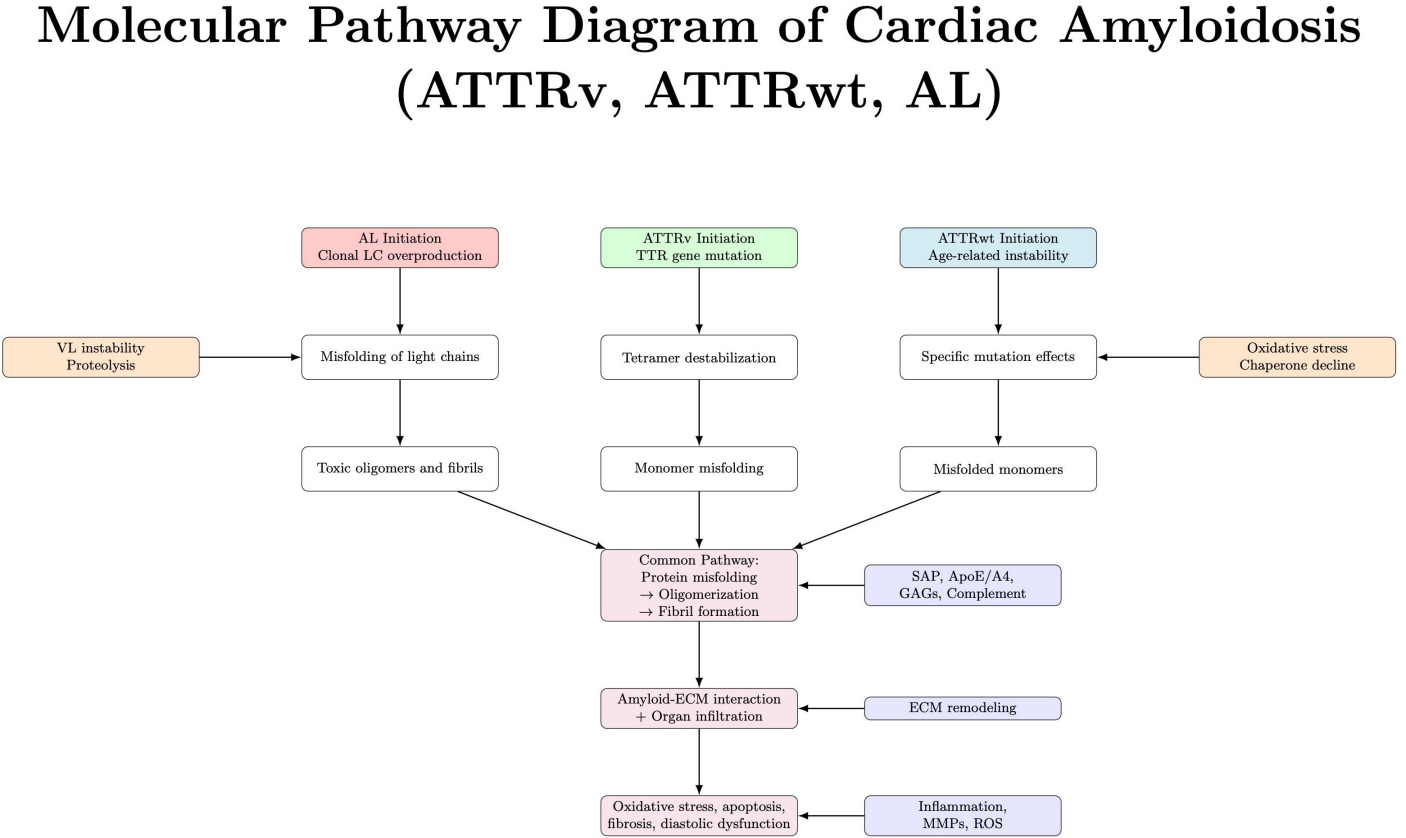
**Molecular mechanism illustrating the convergence of AL, ATTRv, and ATTRwt amyloidosis into a common pathogenic cascade**. Acronyms: SAP, serum amyloid P component; ApoE, apolipoprotein E; GAGs, glycosaminoglycans; ECM, extracellular matrix; MMPs, matrix metalloproteinases; ROS, reactive oxygen species.

Cardiac amyloidosis can be conceptualized as a single disease entity with multiple upstream origins, ATTRv, ATTRwt, and AL, each initiating amyloidogenesis through distinct molecular triggers. Despite these differences, all subtypes converge on a final common pathway marked by extracellular fibril deposition, mitochondrial dysfunction, myocardial structural disruption, oxidative stress, and progressive heart failure, as shown in Fig. 1.

The process begins with the destabilization of native protein conformations. In ATTRv, autosomal dominant mutations in the transthyretin (*TTR*) gene, such as *Val122Ile*, *Thr60Ala*, or *L55P*, destabilize the native TTR tetramer, promoting dissociation into monomers. These monomers are structurally unstable, prone to misfolding into β-sheet–rich conformations that self-assemble via nucleation-dependent polymerization. Proteolytic cleavage, particularly of the C-terminal fragment (residues 49–127), further enhances aggregation potential and yields type A fibrils, shorter, more cardiotropic variants associated with severe phenotypes [52, 55, 56].

In ATTRwt, similar tetramer dissociation occurs not due to genetic mutation but as a consequence of age-related oxidative modifications, impaired hepatic chaperone activity, and environmental stressors. Wild-type TTR also undergoes proteolysis and deposits preferentially in the myocardium as type A fibrils, highlighting a mechanistic overlap with mutant forms [57].

In contrast, AL amyloidosis originates from clonal plasma cell disorders that overproduce immunoglobulin light chains, especially of the λ isotype. These light chains, particularly unstable in the variable domain, undergo sequential unfolding, dimerization, and β-sheet conversion. Cardiomyocyte internalization of prefibrillar light chains triggers p38 MAPK activation, mitochondrial injury, calcium dysregulation, and ROS generation. Meanwhile, fibrillar light chains disrupt extracellular redox signaling and provoke matrix remodeling through metalloproteinase activation (e.g., MMP9, TIMP-1 imbalance) [52, 55, 56].

As aggregation proceeds in all forms, insoluble fibrils accumulate in the myocardial interstitium. These fibrils, composed of TTR or light chains, form rigid cross–β-sheet structures and incorporate accessory molecules such as serum amyloid P, apolipoproteins, glycosaminoglycans, and complement proteins. The cumulative effect is disruption of the extracellular matrix, compression of the microvasculature, and progressive impairment of both diastolic and systolic function [52, 57].

Importantly, cytotoxic oligomeric intermediates exert damage well before fibrils are detectable, highlighting that toxicity precedes and exceeds mere physical infiltration. This explains why early biomarker changes may outpace imaging findings, and why molecular interventions targeting tetramer stabilization or oligomer suppression offer therapeutic potential, even before overt cardiac remodeling becomes evident [55, 58].

## 4. Diagnostic Framework & Imaging Strategies

Non-invasive imaging has reshaped the diagnostic approach to CA, supporting earlier detection, subtype identification, and prognostication without routine reliance on endomyocardial biopsy [54, 55]. Techniques such as echocardiography with strain imaging, cardiac magnetic resonance (CMR), nuclear scintigraphy using 99mTc-labeled tracers, and positron emission tomography (PET) now form the core of diagnostic strategies. These modalities are being enhanced by machine learning approaches that aim to increase accuracy and reduce diagnostic delays [59].

### 4.1 Electrocardiography (ECG)

ECG is a rapid, widely available tool that offers supportive diagnostic clues in cardiac amyloidosis. Classic findings include low QRS voltage, especially in limb leads, and pseudo-infarct patterns, Q waves in the absence of coronary artery disease. Voltage-mass discordance, characterized by increased LV wall thickness on echocardiography despite low ECG voltages, raises suspicion for CA. While low voltage alone has limited sensitivity (46–70%) depending on different ECG criteria used [60], its specificity improves significantly when combined with echo findings such as apical sparing or thickened ventricular walls [61].

The diagnostic performance of ECG increases when integrated with AI-based algorithms. Grogan *et al*. (2021) [59] demonstrated that AI-enhanced ECG analysis achieved an AUC of 0.91 and a positive predictive value of 0.86 in detecting either type of CA. These models can detect subtle waveform features missed by visual inspection, potentially identifying disease earlier in asymptomatic individuals. Moreover, ECG can monitor progression, particularly in AL amyloidosis, where increasing conduction abnormalities and arrhythmias may reflect advancing infiltration [62]. Combining ECG with Echo strain data or integrating it with machine learning models could significantly improve diagnostic accuracy and is a non-invasive, cost-effective screening method that can be particularly useful in resource-limited settings.

### 4.2 Transthoracic Echocardiography

Transthoracic echocardiography (TTE) remains a foundational diagnostic tool in the evaluation of cardiac amyloidosis. Its accessibility and ability to assess structural and functional cardiac abnormalities make it a frontline modality [14, 53, 63]. Typical findings include concentric left ventricular (LV) wall thickening (usually >12 mm), biatrial enlargement, restrictive filling patterns, and mild pericardial effusions. Among these, the presence of an “apical sparing” pattern on global longitudinal strain (GLS) is particularly indicative of cardiac amyloidosis. This pattern, where the apex retains relatively preserved longitudinal strain compared to severely reduced basal and mid-segment strain, has been shown to differentiate CA from other hypertrophic conditions with high accuracy. The relative apical sparing pattern has a reported sensitivity of 93% and specificity of 82% for CA, making it one of the most reliable single echocardiographic features [63, 64].

Strain imaging not only aids in diagnosis but also correlates with functional status and prognosis. Studies have demonstrated that reduced global longitudinal strain correlates with higher amyloid burden and poorer survival [65, 66, 67]. Additional advanced parameters, such as myocardial work indices and non-invasive pressure-strain loops, are under investigation for earlier disease recognition and monitoring therapy response. Furthermore, specific septal wall involvement may aid in subtype differentiation, with one study noting that AL amyloidosis shows more symmetrical wall thickening, while ATTR amyloidosis tends to involve more asymmetrical septal wall thickening demonstrating either a sigmoid shape or reverse septal curvature [64, 68]. Although not definitive, these anatomical differences can support non-invasive typing when integrated with clinical and laboratory data.

### 4.3 Cardiac MRI (CMR)

**Fig. 2.**
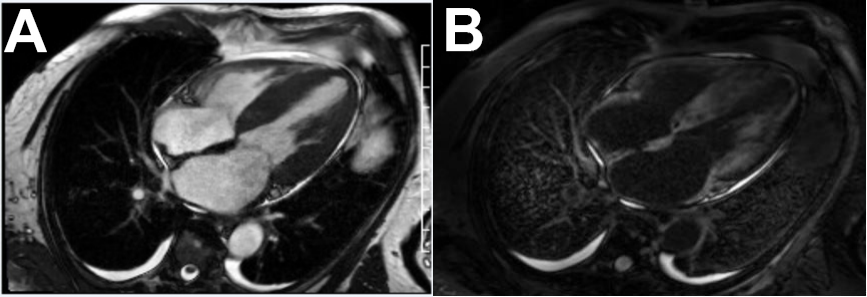
**Multimodality cardiac MRI**. (A) SSFP standstill showing severe concentric LVH, bilateral atrial enlargement, interatrial septal thickening, trace circumferential pericardial effusion. (B) Late Gadolinium enhancing showing diffuse patchy non ischemic pattern.

CMR is one of the most powerful imaging techniques for characterizing myocardial tissue in cardiac amyloidosis. It provides a high-resolution assessment of wall thickness, chamber size, and functional indices, while offering unique capabilities to identify amyloid infiltration. The hallmark CMR feature of CA is late gadolinium enhancement (LGE) with a diffuse subendocardial or transmural pattern (Fig. 2). This enhancement occurs due to expanded extracellular space caused by amyloid deposits. In advanced disease, nulling of the myocardium becomes difficult due to diffuse gadolinium uptake. Sensitivity and specificity of LGE for CA are approximately 93% and 87%, respectively [69, 70].

**Fig. 3.**
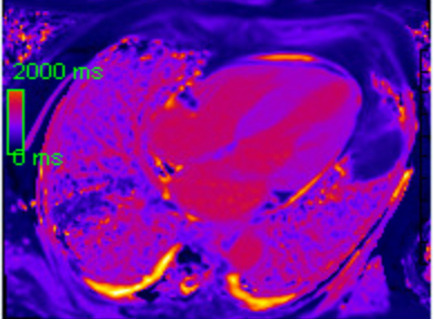
**Parametric mapping with elevated native T1 (Mid anterior septal value 1186 ms, ECV calculated 65%)**.

T1 mapping and extracellular volume (ECV) quantification add further diagnostic and prognostic value (Fig. 3). ECV values greater than 40% are strongly associated with CA and correlate with amyloid burden. These metrics not only support diagnosis but also allow tracking of disease progression and response to therapy. Pan *et al*. (2020) [71] showed that ECV outperformed LGE through diagnostic odds ratio of cardiac amyloidosis and had an overall higher hazard native for adverse events in comparison to LGE and native T1. CMR is especially valuable in AL CA, where nuclear imaging may be negative. It also assists in distinguishing CA from other hypertrophic conditions like hypertrophic cardiomyopathy or hypertensive heart disease, using both functional and tissue-based parameters. Although gadolinium use limits its utility in advanced renal dysfunction, non-contrast T1 mapping approaches are under development to extend access to a broader patient population.

### 4.4 Nuclear Scintigraphy (99mTc-PYP)

Nuclear scintigraphy with technetium-labeled bone tracers, particularly 99mTc-pyrophosphate (PYP), is a cornerstone in the non-invasive diagnosis of transthyretin (ATTR) cardiac amyloidosis. It relies on the affinity of bone-seeking tracers for amyloid fibrils deposited in myocardial tissue. When performed appropriately, 99mTc-PYP imaging allows for diagnosis without the need for endomyocardial biopsy, provided monoclonal gammopathy is excluded.

The test involves planar and SPECT imaging, typically 1–3 hours post-injection. Myocardial uptake is visually graded on the Perugini scale from 0 to 3. Grades 2 or 3 (referring to moderate to high uptake relative to bone) in the absence of monoclonal proteins are considered diagnostic of ATTR amyloidosis, with reported specificity and positive predictive value of 100%, but a sensitivity of 70% only in the absence of monoclonal gammopathy [72, 73]. False negatives may occur in early disease. Quantitative uptake can also be assessed using the heart-to-contralateral lung (H/CL) ratio, where a value ≥1.5 at 1 hour supports the diagnosis of ATTR amyloidosis [74]. False positives may occur in AL amyloidosis and with certain technical factors, highlighting the importance of appropriate patient selection and adjunct serologic testing. Scintigraphy is less reliable in early-stage disease, where myocardial amyloid deposition may be insufficient for tracer uptake, and in patients with concurrent monoclonal gammopathies. Despite these limitations, its accessibility and accuracy have made it a front-line test for suspected ATTR-CA.

### 4.5 Positron Emission Tomography (PET)

PET imaging offers high-resolution, molecular-level assessment of myocardial amyloid burden using radiotracers that bind specifically to amyloid fibrils. Commonly used tracers include 18F-florbetapir, 18F-flutemetamol, and 11C-Pittsburgh compound B (PiB). These agents have been validated for imaging cerebral amyloidosis and are now being investigated for cardiac applications, especially in suspected AL amyloidosis, were scintigraphy lacks sensitivity.

PET identifies myocardial uptake by comparing early and delayed images and using retention indices or standardized uptake values (SUVs). Reported sensitivities for PET tracers in cardiac amyloidosis range from 80–90%, with specificity close to 100% in AL subtypes [75]. Unlike scintigraphy, PET may detect amyloid deposits earlier in the disease course and provides the potential to quantify disease burden and monitor therapy response. However, limitations include limited availability, high cost, and lack of FDA-approved tracers specifically for cardiac amyloid imaging. Research continues to refine its role, particularly in combination with other modalities such as CMR or PYP, where discordant findings may benefit from PET clarification. PET use remains largely investigational.

### 4.6 Combined Diagnostic Modalities

**Fig. 4.**
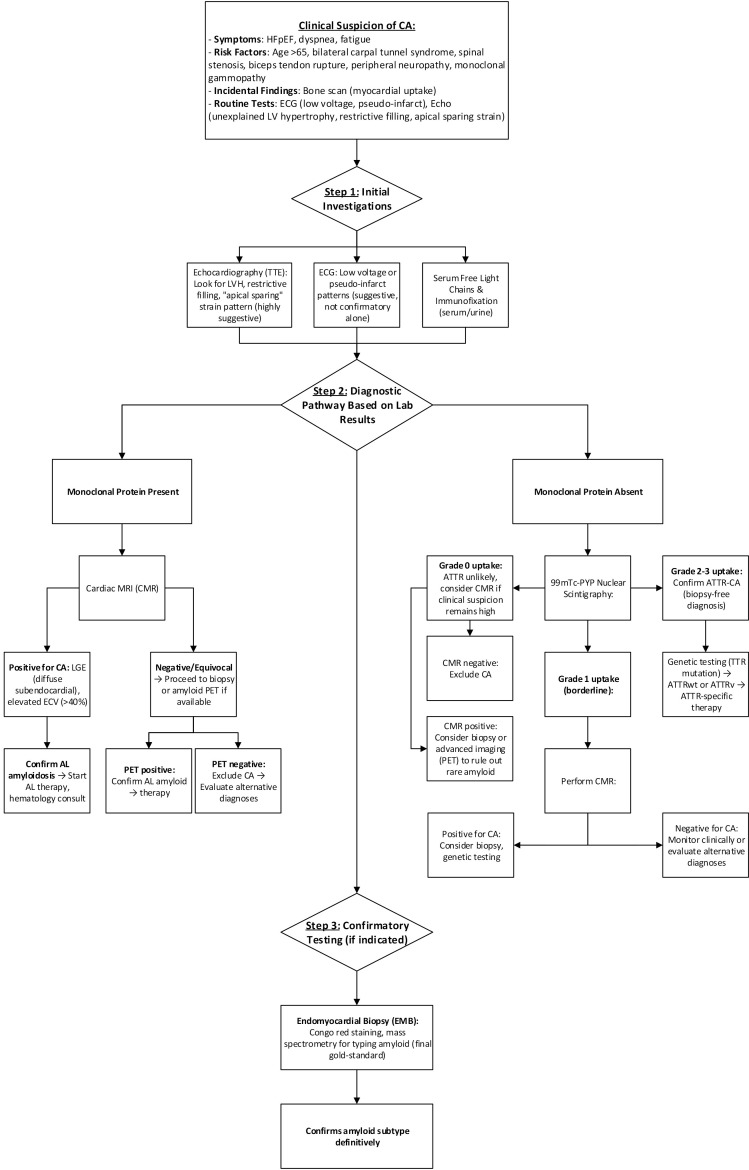
**Proposed diagnostic algorithm for CA in a resource-sufficient setting**.

Integrating modalities improves diagnostic precision. Echo and ECG together enhance specificity to 91% [60]. AI-assisted ECG algorithms combined with standard imaging yield an AUC of 0.91, noting a PPV of 0.86 for either type of CA [59]. Machine learning applied to CMR enhances amyloid subtype classification and burden estimation. Combining nuclear scintigraphy (like SPECT) with PET may improve characterization in early or borderline ATTR cases and possibly improve the ability to differentiate between ATTR and AL amyloidosis. Modality selection should be based on clinical context, laboratory markers, and test availability. ATTR Amyloidosis is best identified by 99mTc-PYP imaging (Perugini grade ≥2 without monoclonal protein), supported by echo findings of apical sparing and basal septal thickening [72, 73]. Whereas AL Amyloidosis is effectively evaluated using CMR (LGE and ECV) and PET tracers. Confirmation with serum free light chains is essential [51, 59, 71]. Suspected cases begin with clinical assessment and baseline ECG and echocardiography. If imaging findings are suggestive, serum and urine immunofixation and free light chain testing are performed. If monoclonal proteins are absent, a 99mTc-PYP scan can confirm ATTR amyloidosis. If present, CMR or PET may confirm AL type. However, indeterminate results may still require biopsy, as it is still considered the gold standard diagnostic tool for CA. A proposed algorithm is detailed in Fig. 4.

### 4.7 Right Ventricular Involvement in Cardiac Amyloidosis: A Critical but Underrecognized Prognostic and Diagnostic Marker

CA affects both ventricles, with right ventricular (RV) involvement linked to worse outcomes. Recent imaging studies have highlighted the prognostic significance of right-sided involvement in CA, a dimension often underrecognized in traditional assessments [76]. In AL amyloidosis, right ventricular (RV) involvement is a strong negative prognostic factor. RV longitudinal strain >–17% and elevated NT-proBNP levels independently predict mortality. RV dysfunction typically follows left ventricular changes and indicates advanced disease. Routine RV strain assessment improves risk stratification and guides timely intervention in affected patients [77].

Furthermore, differentiating CA from hypertrophic cardiomyopathy (HCM) is clinically important due to overlapping features. Advanced cardiac imaging using right ventricular strain parameters offers diagnostic value. In a comparative study, effective differentiation of CA from HCM was achieved using right ventricular global longitudinal strain (GLS: 16.5 ± 3.9% vs. –21.3 ± 6.7%, *p* = 0.032), Global Radial Strain (global radial strain, GRS: 11.7 ± 5.3% vs. 16.5 ± 7.1%, *p* < 0.001), and circumferential strain (GCS: –7.6 ± 4.0% vs. –9.4 ± 4.4%, *p* = 0.015). Among these, GRS provided the highest diagnostic accuracy (area under curve (AUC) = 0.86). Furthermore, binomial regression analysis identified right ventricular ejection fraction (RV-EF, *p* = 0.017) as a significant predictor for distinguishing CA from HCM. Notably, RV strain values were similar across different CA subtypes, reinforcing the utility of RV functional assessment in diagnostic stratification [78]. Incorporating right-sided parameters into diagnostic and prognostic frameworks could enhance risk stratification and guide therapeutic decision-making. These findings argue for a more holistic assessment of cardiac function in CA, integrating both ventricles to better diagnose CA and reflect outcomes.

### 4.8 CA Diagnostic Challenges in Low Socioeconomic Areas

**​**In resource-limited settings, diagnosing CA presents significant challenges due to the unavailability of advanced diagnostic tools such as CMR, nuclear scintigraphy, and endomyocardial biopsy. As such, clinicians often rely on basic evaluations, including clinical history, ECG, and echocardiography, to raise suspicion for CA. However, these modalities lack specificity, leading to frequent misdiagnoses and delayed treatment initiation. A study from Ethiopia emphasized the pivotal role of ECG and echocardiography in diagnosing CA in resource-constrained environments, highlighting the need for heightened clinical suspicion and the utilization of available tools to improve outcomes [79]. ​In addition, the use of telemedicine consultations could also help bridge gaps in expertise and provide quicker access to specialized care.

Despite these efforts, there is a notable absence of published diagnostic algorithms tailored specifically for low-resource settings. This gap emphasizes the necessity for developing cost-effective, accessible diagnostic pathways that can be implemented in underserved regions. The lack of such algorithms may contribute to delays in diagnosis, particularly among vulnerable populations. Shankar *et al*. [80] reported that Black patients with transthyretin amyloid cardiomyopathy (ATTR-CM) were disproportionately represented in the most socioeconomically deprived categories and experienced higher rates of heart failure hospitalization or death over five years compared to White patients, independent of disease stage at diagnosis. These findings highlight the urgent need for equitable diagnostic strategies to mitigate disparities and improve outcomes in low socioeconomic populations.

**Fig. 5.**
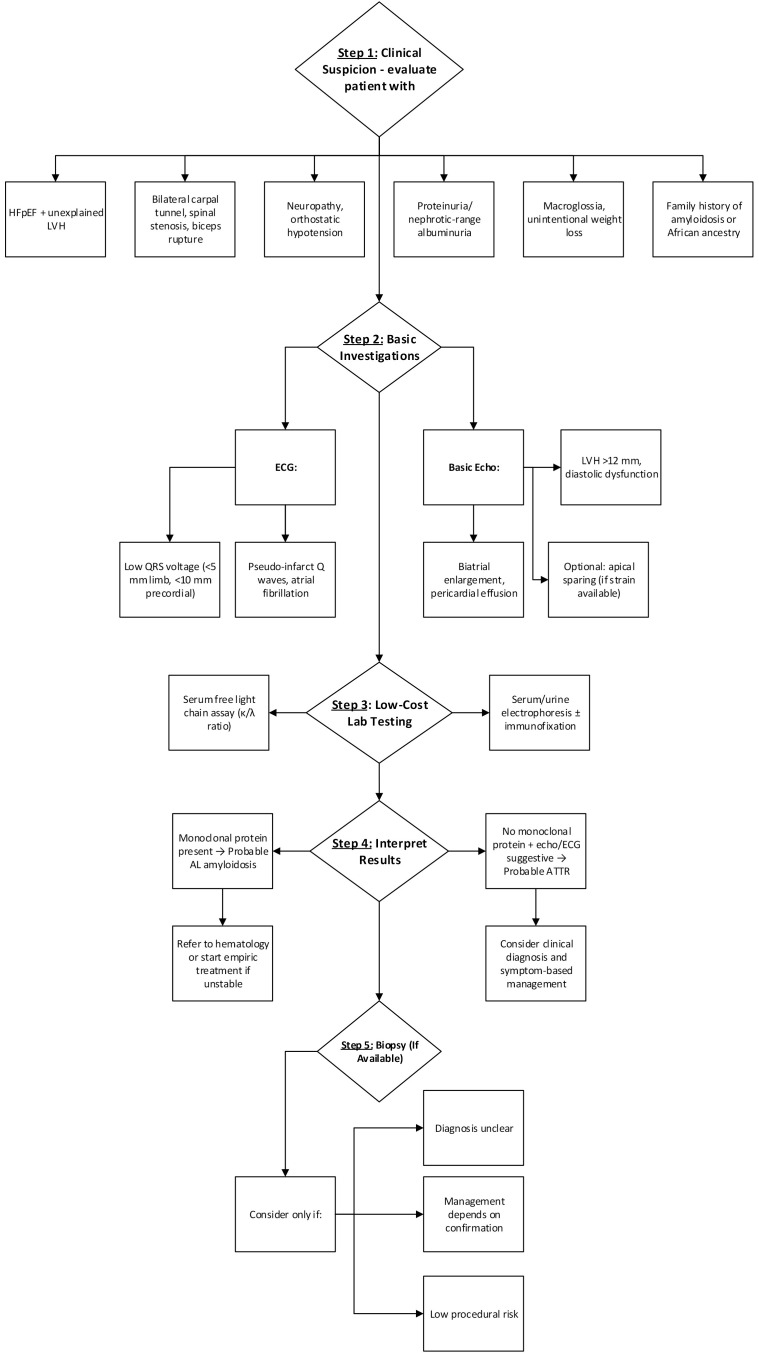
**Proposed cardiac amyloidosis diagnostic algorithm in low-socioeconomic areas**.

Therefore, an alternative diagnostic algorithm for low socioeconomic communities is a must. In resource-limited environments, a practical approach involves initial screening with ECG and clinical history. Basic echocardiography and serum free light chain analysis can guide suspicion. Where imaging is inaccessible, clinical features and lab work may direct the decision to biopsy, as EMB remains the gold standard in CA diagnosis. A proposed cost-effective algorithm described in Fig. 5 prioritizing echo and ECG integration can help identify cases earlier in underserved populations.

### 4.9 AI Integration Into Current Diagnostic Modalities

The integration of artificial intelligence (AI) into existing diagnostic modalities has significantly enhanced the detection and characterization of CA. In electrocardiography, AI-enhanced algorithms have demonstrated high diagnostic performance, with sensitivity and specificity rates exceeding 90% in detecting CA, outperforming traditional ECG interpretations [59]. Echocardiography has similarly benefited from AI, where machine learning models analyzing parameters like global longitudinal strain and apical sparing patterns have achieved higher diagnostic accuracies, surpassing conventional assessments, and providing particular benefit at reducing human variability [81, 82]. CMR has seen the application of deep learning models, particularly convolutional neural networks (CNNs), which have achieved AUC values of 0.96, with sensitivities and specificities around 94% and 90%, respectively [83]. Furthermore, AI applications in nuclear imaging, such as bone scintigraphy, have improved the differentiation between CA subtypes, aiding in more precise diagnoses through the identification of subtle patterns and features that may be missed through human interpretation, and potentially serve as a screening tool for ATTR-CA [84]. These advancements underscore the potential of AI to augment diagnostic accuracy, reduce inter-observer variability, and facilitate earlier detection of CA, especially in settings where access to specialized expertise may be limited.

## 5. Histopathologic and Biomarker-Guided Strategies in Diagnosis and Prognostication

CA represents a prototypical infiltrative cardiomyopathy wherein misfolded protein fibrils deposit extracellularly within the myocardium, leading to progressive ventricular stiffness, arrhythmias, and eventual heart failure [85]. Diagnosing CA with accuracy and timeliness necessitates integration of histopathologic evaluation, definitive tissue typing, and biomarker-based assessment. The distinguishing histologic features of amyloid infiltration, combined with advanced diagnostic modalities, including Congo red staining, immunohistochemistry, and mass spectrometry, form the backbone of tissue-based diagnosis [54]. Simultaneously, circulating biomarkers such as NT-proBNP and cardiac troponins provide critical insights into myocardial stress and injury, contributing to both diagnostic suspicion and longitudinal risk stratification [86, 87].

### 5.1 Tissue Characterization

Histopathologically, cardiac amyloid deposition is characterized by the extracellular accumulation of eosinophilic, amorphous material that exhibits apple-green birefringence under polarized light following Congo red staining [88]. This deposition, which disrupts myocardial architecture and ensheathes cardiomyocytes and intramyocardial vessels, distinguishes CA from other restrictive cardiomyopathies (RCMs). Unlike inflammatory or metabolic causes of RCM, such as sarcoidosis, Fabry disease, hemochromatosis, or endomyocardial fibrosis, amyloid infiltration lacks significant inflammatory response, intracellular storage, or organized fibrous tissue patterns [89]. For instance, sarcoidosis features non-caseating granulomas; Fabry disease manifests intracellular glycosphingolipid vacuoles; hemochromatosis presents with intracellular iron granules; and radiation-induced fibrosis demonstrates dense, acellular collagen bands without Congo red positivity [90]. Each alternative diagnosis carries unique histochemical and ultrastructural signatures, which are essential to differentiate from CA and guide appropriate therapeutic strategies.

Tissue characterization of CA is initiated through Congo red staining, which remains a foundational technique due to its ability to universally detect amyloid fibrils regardless of precursor protein. However, limitations in sensitivity, particularly in sparse or suboptimally processed specimens, necessitate supplemental diagnostic tools [88, 91]. Immunohistochemistry (IHC) can help subtype amyloid by targeting κ and λ light chains or transthyretin (TTR), aiding in the differentiation between AL (light chain) and ATTR (transthyretin) amyloidosis [91, 92]. Yet, the limitations of IHC, chiefly antibody cross-reactivity, epitope masking, and technical variability, compel the use of mass spectrometry (MS)-based proteomic analysis in uncertain cases. MS provides definitive identification of amyloid subtype through high-resolution peptide profiling and is now considered the diagnostic gold standard, particularly in complex or ambiguous scenarios [93]. Transmission electron microscopy (TEM) and confocal laser scanning microscopy further enhance diagnostic precision in equivocal cases, enabling visualization of characteristic 10-nm fibrils or dual-fluorescent co-localization, respectively [94].

Endomyocardial biopsy (EMB) remains the definitive diagnostic procedure for CA, offering near-complete sensitivity when multiple right ventricular septal samples are obtained and appropriately processed [95, 96]. The pattern of amyloid deposition, whether diffuse interstitial, nodular, vascular, or endocardial, can offer preliminary clues to the amyloid subtype. AL amyloidosis tends to show diffuse infiltration and pronounced vascular involvement, whereas ATTR often presents with nodular or subendocardial patterns and a patchier distribution [97]. However, histologic pattern alone is insufficient for subtype determination, necessitating confirmatory immunophenotyping and, ideally, proteomic analysis.

Sampling strategy in EMB is critically important. While non-cardiac tissue biopsies, such as abdominal fat pad, bone marrow, or renal biopsies, can yield diagnostic amyloid in AL cases, they are notably less sensitive for ATTR, particularly the wild-type form [98, 99]. Negative results from surrogate sites in patients with suspected cardiac involvement should prompt EMB, especially when clinical or imaging findings suggest CA. In recent years, technetium-labeled bone scintigraphy has enabled noninvasive diagnosis of ATTR amyloidosis when combined with negative serum and urine studies for monoclonal protein [100]. Nevertheless, EMB remains essential in cases with discordant findings or dual pathologies (e.g., concurrent monoclonal gammopathy and positive PYP scan), as misclassification could lead to inappropriate therapy.

Following confirmation of amyloid presence in EMB tissue, immunophenotyping and proteomic confirmation are paramount. Positive Congo red staining is followed by targeted IHC for κ, λ, and TTR. In cases of IHC ambiguity or when high-stakes therapeutic decisions are involved, MS is deployed for definitive fibril protein identification. This is especially vital in differentiating AL from ATTR, as therapeutic implications are profound: AL necessitates immediate initiation of chemotherapy (often involving proteasome inhibitors, immunomodulators, and anti-CD38 monoclonal antibodies), while ATTR is treated with TTR stabilizers (e.g., tafamidis) or gene-silencing therapies, and supportive measures [54, 85, 91].

Biopsy results further inform disease burden and treatment urgency. Extensive interstitial involvement or high amyloid burden in EMB correlates with advanced disease stage and poor prognosis, guiding decisions about the aggressiveness of therapy or need for advanced heart failure interventions, including transplantation. The presence of AL amyloid on EMB, even in the absence of systemic signs, warrants systemic evaluation and prompt therapy, given the high mortality risk associated with cardiac AL [97].

### 5.2 Biomarker-Guided Prognosis & Limitations

Complementing tissue-based diagnostics, circulating biomarkers provide essential functional and prognostic information. NT-proBNP and cardiac troponins serve as key indicators of myocardial stress and injury, respectively. In AL amyloidosis, light chains exhibit direct cardiotoxicity in addition to promoting diastolic dysfunction, resulting in disproportionately elevated NT-proBNP and troponin levels compared to ATTR for similar degrees of hypertrophy. This biomarker profile reflects both hemodynamic compromise and cellular injury, offering insight into disease severity and progression [86, 87].

NT-proBNP and troponin levels form the basis of established staging systems. In AL amyloidosis, the Mayo Clinic staging system classifies patients into stages I–III (and later IV with free light chain inclusion) based on NT-proBNP (>332 pg/mL) and troponin T (>0.035 ng/mL) thresholds, correlating strongly with survival outcomes. In ATTR amyloidosis, Gillmore *et al*. [101] proposed a parallel staging system utilizing NT-proBNP (>3000 pg/mL) and eGFR (<45 mL/min), reflecting the predominance of hemodynamic stress and age-related renal dysfunction in this population. These staging tools not only inform prognosis but also aid in therapeutic decision-making and patient counseling [86, 87].

Importantly, serial measurement of NT-proBNP is employed to monitor treatment response, particularly in AL amyloidosis. A reduction of >30% and >300 pg/mL from baseline (provided baseline >650 pg/mL) defines a cardiac response, which correlates with improved survival. In ATTR, the biomarker response is slower and less dramatic, but stabilization or attenuation of NT-proBNP rise is considered indicative of therapeutic efficacy. Troponin, while less dynamic, can serve as a supplemental marker of myocardial injury, and sustained elevation often portends ongoing damage [86, 87, 102].

Despite their utility, these biomarkers have inherent limitations. Both are renally cleared and subject to fluctuation with volume status, atrial arrhythmias, or assay variability. Elevated levels are not specific to amyloidosis and must be interpreted in the clinical context. Nevertheless, their integration into amyloidosis management has markedly enhanced clinicians’ ability to stage disease, monitor therapy, and refine prognosis [102].

## 6. Clinical Implications: Accurate Subtype Diagnosis Directs Therapy

Precise diagnosis of cardiac amyloidosis is not only of academic interest but directly impacts patient outcomes. Distinguishing between light-chain (AL) and transthyretin (ATTR) amyloidosis is essential because effective disease-modifying therapies are now available and are subtype-specific [8, 103, 104, 105].

In AL amyloidosis, plasma cell–directed therapy should begin promptly to suppress amyloidogenic light chains. Bortezomib-based combinations with cyclophosphamide and dexamethasone, with or without daratumumab, improve hematologic and organ responses and survival [8, 103]. Autologous stem cell transplantation is appropriate for selected patients and can yield durable remission [106].

Whereas in ATTR amyloidosis, Tafamidis stabilizes transthyretin and reduces all-cause mortality and cardiovascular hospitalizations in transthyretin cardiomyopathy [104]. Gene-silencing therapies such as patisiran and inotersen are approved for hereditary ATTR polyneuropathy and show cardiac benefits in dedicated and exploratory analyses, with cardiomyopathy-focused trials ongoing [105, 107]. Next-generation stabilizers including acoramidis show promising phase 3 results and may broaden options pending regulatory decisions. Supportive care parallels AL with frequent need for diuretic optimization and prudent arrhythmia control [108]. Early-phase trials of CRISPR-based gene editing (e.g., NTLA-2001) have shown promising results in reducing transthyretin levels, but this approach remains investigational at present [109].

## 7. Limitations

This review has several key limitations. Much of the literature cited relies on observational or retrospective data, which constrains the ability to establish causal relationships. The clinical heterogeneity between amyloidosis subtypes, particularly AL and ATTR, challenges the adoption of unified diagnostic or prognostic models. Although we included the most current data available at the time of writing, newly emerging diagnostics and therapies may not yet be reflected. Finally, artificial intelligence tools (ChatGPT-4o, QuillBot, Grammarly, EndNote) were used to enhance grammar and structural flow; however, all scientific content was independently drafted, validated, and is the sole responsibility of the authors.

## 8. Conclusion

CA exemplifies how a deeper understanding of molecular pathogenesis can transform clinical paradigms. This review has detailed how CA arises from the aggregation of misfolded proteins, either transthyretin or immunoglobulin light chains, into amyloid fibrils that accumulate in myocardial tissue. These deposits compromise myocardial function not only through extracellular infiltration but also by directly inducing cytotoxicity, oxidative stress, and cardiomyocyte apoptosis. Importantly, while distinct in their origins, all forms of CA converge on shared pathophysiologic endpoints, including mitochondrial dysfunction and progressive structural remodeling of the heart.

Histopathologic assessment remains central to definitive diagnosis, yet non-invasive tools have reshaped the diagnostic landscape. Techniques such as echocardiographic strain imaging, cardiac magnetic resonance with tissue characterization, and bone scintigraphy offer powerful, accessible methods for earlier and more accurate detection of CA subtypes. The addition of serum biomarkers like NT-proBNP and troponin enhances both diagnostic sensitivity and risk stratification, supporting their integration into staging systems that guide clinical management.

Despite these advances, challenges remain, particularly in resource-limited settings where access to specialized imaging and histopathology may be constrained. Here, simplified algorithms incorporating basic ECG, echocardiography, and clinical pattern recognition may aid earlier identification. Moreover, disparities in diagnosis across sex, ethnicity, and socioeconomic lines underscore the importance of equitable diagnostic strategies.

Ultimately, recognizing CA as a mechanistically distinct and diagnostically nuanced disease enables clinicians to intervene earlier and more precisely. As diagnostic capabilities and molecular insights continue to evolve, so too will opportunities to improve outcomes for patients facing this historically neglected but increasingly treatable condition.
